# Prognostic value of lymphocyte-to-monocyte ratio in gastric cancer patients treated with immune checkpoint inhibitors: a systematic review and meta-analysis

**DOI:** 10.3389/fimmu.2023.1321584

**Published:** 2023-11-27

**Authors:** Pingping Mei, Wenzhe Feng, Yanrong Zhan, Xiutian Guo

**Affiliations:** ^1^ Shanghai Municipal Hospital of Traditional Chinese Medicine, Shanghai University of Traditional Chinese Medicine, Shanghai, China; ^2^ Shaanxi University of Chinese Medicine, Xianyang, Shaanxi, China

**Keywords:** lymphocyte-to-monocyte ratio, gastric cancer, immune checkpoint inhibitors, prognostic value of survival, meta-analysis

## Abstract

**Background:**

Emerging evidence suggests a correlation between the lymphocyte-monocyte ratio (LMR) and the prognosis in patients with gastric cancer (GC) undergoing immune checkpoint inhibitor (ICI) therapy. Nevertheless, the existing findings remain contentious.

**Methods:**

A comprehensive search of literature was conducted in databases including PubMed, Embase, Web of Science, and the Cochrane Library, spanning from the inception of each database to August 30, 2023 to collect studies exploring the interplay between LMR and clinical outcomes. Eligible studies were selected following predefined inclusion and exclusion criteria. Primary outcomes encompassed progression-free survival (PFS) and overall survival (OS), which were estimated using hazard ratios (HR) and corresponding 95% confidence intervals (CI).

**Results:**

Our analysis incorporated eight cohort studies, involving 815 patients. Aggregate data revealed associations between an elevated LMR at baseline and prolonged PFS (HR=0.58; 95% CI: 0.47–0.71, p<0.00001) and improved OS (HR=0.51, 95% CI: 0.33–0.79; p=0.003). Furthermore, LMR exhibited a favorable association with PFS after treatment (HR=0.48; 95% CI: 0.29–0.79; p= 0.004), while such a correlation was not evident in the OS analysis. Importantly, a high level of LMR was associated with prolonged PFS across varying sample sizes, follow-up duration, treatment combinations, line of therapy, and cut-off values.

**Conclusion:**

A high pre-treatment LMR is associated with improved OS and PFS in GC patients treated with ICIs. LMR emerges as a potent biomarker for prognostic assessment in these patients, offering valuable insights for informed treatment decisions within the domain of GC immunotherapy.

**Systematic review registration:**

PROSPERO, identifier CRD42021228512

## 1 Introduction

Despite the decline in the incidence of gastric cancer (GC) in recent decades, it continues to pose a substantial global health challenge, standing as the fifth most prevalent malignancy globally and the third foremost cause of mortality related to cancer, especially in East Asia (1). Recent years have witnessed a transformative shift in cancer treatment with the introduction of immune checkpoint inhibitors (ICIs), such as monoclonal antibodies targeting programmed cell death ligand-1 (PD-L1) and programmed cell death-1 (PD-1). These ICIs suppress and harness the immune checkpoint pathway to combat cancer cells (2–4). Noteworthy among these advancements is pembrolizumab, a PD-1 inhibitor, which has shown substantial efficacy as a standalone treatment for advanced gastric cancer patients who have undergone at least two prior therapeutic approaches, achieving an objective response rate (ORR) of 11.6% (95% confidence interval [CI]: 8.0%–16.1%) (5). Furthermore, a study exhibited a notable improvement in survival rates when comparing nivolumab, another PD-1 inhibitor, to a placebo (HR=0.63, 95% CI: 0.51–0.78) among patients with advanced gastric cancer who had received two or more lines of therapy (6). However, it is crucial to recognize that although ICIs offer enduring anti-tumor effects, they also come with the potential for severe toxicity and substantial treatment costs. Therefore, the identification of the patients most likely to benefit from ICI therapy holds paramount importance (7). Nevertheless, the pursuit of effective biomarkers capable of predicting immunotherapy outcomes remains a challenge in contemporary clinical practice.

It is increasingly acknowledged that tumor development is influenced not only by tumor-specific factors but also by the host’s immune status, as the systemic inflammatory response of the host is essential for processes like tumor development, angiogenesis, and disease progression (8–10). Systemic inflammation can be evaluated by examining changes in the cellular composition of the peripheral blood, including lymphocytes, monocytes, neutrophils, and platelets. through metrics such as the systemic immunoinflammatory index (SII), the platelet-lymphocyte ratio (PLR), and the lymphocyte-monocyte ratio (LMR) (11). Numerous studies have established significant correlations between these biomarkers and survival in various malignant tumors. For instance, elevated PLR, along with reduced LMR, have consistently been associated with poorer prognosis in conditions like lung cancer, colorectal cancer, and melanoma (12–14). Additionally, several investigations have confirmed the significant prognostic role of LMR in predicting adverse survival outcomes in GC patients who have undergone radical resection or chemotherapy (15, 16). Nonetheless, the utilization of LMRs in the context of GC immunotherapy remains an area that requires further exploration.

While meta-analyses have been published examining the impact of LMR on the prognosis of GC patients who have undergone curative resection (17), no comprehensive reviews have been conducted to assess the prognostic impact of LMR in GC patients who received ICI treatment to date. Consequently, this meta-analysis was undertaken to assess the prognostic impact of LMR.

## 2 Materials and methods

### 2.1 Literature search

The reporting of this study followed the guidelines outlined in the Preferred Reporting Items for Systematic Reviews and Meta-Analyses (PRISMA2020) statement (18), and the research protocol was registered on the International Prospective Systematic Evaluation Registry (PROSPERO: CRD42021228512). Two investigators, MPP and ZYR, were responsible for crafting the search strategy. They independently developed subject terms and keywords for the search of multiple databases including PubMed, Embase, Web of Science, and the Cochrane Library, covering the period from the inception of the databases to August 30, 2023. The search used a wide range of terms such as “gastric cancer,” “gastric carcinoma,” “gastric tumor,” “stomach neoplasms,” “stomach tumor,” “Immune Checkpoint Blockade,” “Immune Checkpoint Inhibitor,” “PD-L1 Inhibitors,” “PD 1 Inhibitors,” “Programmed Death-Ligand 1 Inhibitors,” “CTLA-4 Inhibitors,” “Cytotoxic T-Lymphocyte-Associated Protein 4 Inhibitors,” “pembrolizumab,” “nivolumab,” “tremelimumab,” “avelumab,” “sintilimab,” “ipilimumab,” “Lymphocytes,” “Monocytes,” “monocyte lymphocyte ratio(MLR),” and “lymphocyte monocyte ratio(LMR).” **Supplementary Table S1** presents the literature search strategy.

### 2.2 Study selection

Studies eligible for inclusion in our analysis should meet the following criteria: (1) Patients were diagnosed with GC through pathologic observation; (2) ICIs were administered either as a monotherapy or in combination; (3) Studies focused on the assessment of the prognostic impact of LMR on PFS or OS; (4) Studies provided data on the risk ratio (HR) with a 95% confidence interval (CI), which could be either extracted directly from studies or computed based on available data; (5) Patients were categorized into high-LMR and low-LMR groups according to specified cut-off values; (6) Studies have been fully published. In contrast, the exclusion criteria are as follows: (1) Reviews, comments, meeting abstracts case reports, and letters were excluded; (2) Literature lacking sufficient information to compute HR and 95% CI were not considered; (3) Studies not providing survival data were removed; (4) Studies with data that were duplicated or overlapping were excluded.

Two researchers (MPP and ZYR) independently reviewed the titles and abstracts of studies retrieved from databases, downloaded full-text articles, and evaluated them to obtain eligible studies. Any disagreements during the study selection process were resolved through consensus.

### 2.3 Data extraction

The extraction of data was carried out by two researchers, MPP and ZYR, independently. Any disagreements were settled through consensus among all co-authors. Extracted information included the name of the first author, publication year, country (study location), study type, sample size, patient age, study duration, treatment method, specific immune checkpoint inhibitors used, timing of detection, cut-off value, follow-up duration, and HRs (95% CIs) for OS and PFS. It should be noted that, in terms of studies that reported MLR data (19, 20), we took the reciprocal of related HR values and corresponding confidence intervals, and exchanged the upper and lower confidence limits to convert MLR into LMR values, so as to facilitate our statistical analysis.

### 2.4 Quality assessment

We utilized the Newcastle-Ottawa Quality Assessment Scale (NOS) to assess studies incorporated into our meta-analysis, where they were evaluated based on three parameters: selection, comparability, and outcomes, with a maximum score of nine points awarded to a study (21). Studies scoring between 7 and 9 were classified as having high quality (22).

### 2.5 statistical analysis

The pooled HRs with associated 95% CIs were calculated to evaluate the prognostic value of LMR in GC patients treated with ICIs, where MLR correlation results were converted into LMR format as necessary. Cochran’s Q test and Higgins *I* ^2^ statistic were utilized for measuring heterogeneity (23). Subsequently, a random-effects model was employed for data analysis when *I* ^2^ >50%; otherwise, a fixed-effects model was used. Subgroup and sensitivity analyses were conducted to validate the robustness of results related to OS and PFS. To assess the presence of publication bias, we employed funnel plots and conducted Egger’s and Begg’s tests. P<0.05 was set as the threshold for statistical significance. All statistical analyses were carried out using STATA 15.0 and Review Manager 5.4 software.

## 3 Results

### 3.1 Study characteristics

**Figure 1:**
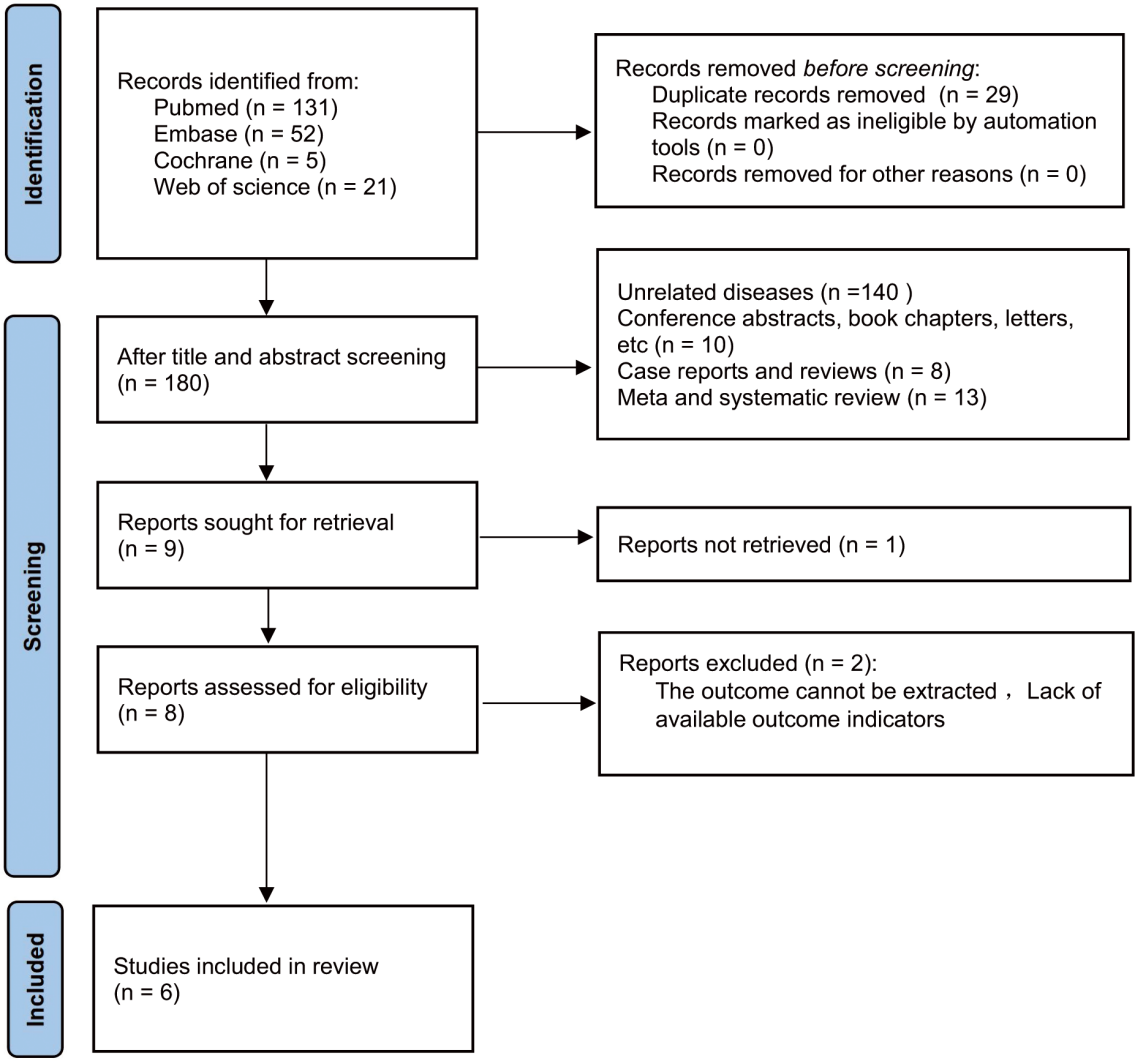
Flow chart of literature screening.

A total of 209 articles were obtained from the initial search of databases. Among them, 29 articles were removed for duplicate publication. Upon reviewing the titles and abstracts of the remaining studies, we excluded 171 studies. The full texts of nine studies were then assessed. Among these, three studies were excluded primarily due to insufficient relevant data required for survival analysis. Ultimately, this meta-analysis included six studies, encompassing a total of 815 patients (19, 20, 24–27) into (**Figure 1**).

Among the eligible six studies, one study was conducted in Japan, while the remaining five were conducted in China. Notably, two of the eligible articles (19, 20) included two cohort studies respectively, resulting in a total of eight cohort studies. Among them, seven cohort studies were retrospective (19, 20, 24, 26, 27), whereas the remaining one was prospective (25). All cohort studies were published in English and released between 2021 and 2022. All studies employed PD-1/PD-L1 antibodies and incorporated two groups for analysis: high-LMR and low-LMR groups. Regarding the measurement of LMR, six studies measured LMR at baseline, one evaluated LMR after treatment, and one examined both baseline and post-treatment LMR. In light of LMR evaluation, eight studies probed into the prognostic implications of LMR on OS, while seven studies delved into its prognostic significance on PFS. **Table 1** presents the included studies’ characteristics.

**Table 1:** Basic characteristics of the included literature.

Author	Year	Sources of patients	Study type	Age	Duration	sample size	Follow-up (month)	ICIs agents	Combined medication	Line of therapy	Test time	Survival analgsis	LMR cut-off	Outcome	Quality score
Chen et al.	2021	China	Cohort(retrospective)	60	2015-2019	139	23.8	Anti-PD-(L)1+ chemo/anti-VEGF/anti-HER/anti-CTLA-4	Monotherapy, combined therapy	1st-line or later	Baseline/posttreatment	Multivariate	3.5	OS,PFS	7
Qu et al.(1)	2022	China	Cohort(retrospective)	NA	2019-2021	53	17.5	Anti-PD-1+ chemo	combined therapy	1st-line	Baseline	Multivariate(OS)/Univariate(PFS)	5	OS,PFS	8
Qu et al.(2)	2022	China	Cohort(retrospective)	NA	2019-2021	53	15.9	Anti-PD-1+ chemo	combined therapy	2nd-line or later	Baseline	Univariate	5	OS,PFS	8
Ruan et al.	2021	China	Cohort(prospective)	60	2016-2017	58	4.5	Toripalimab	Monotherapy	1st-line or later	Baseline	Multivariate(OS)/Univariate(PFS)	2.8	OS,PFS	7
Tokumaru et al.	2021	Japan	Cohort(retrospective)	69	2017-2020	55	NA	Nivolumab	Monotherapy	2nd-line or later	Posttreatment	Multivariate	3.28	OS	8
Wan et al.(1)	2022	China	Cohort(retrospective)	64	2017-2020	45	27.3	Anti-PD-1+ chemo	combined therapy	1st-line	Baseline	Univariate	2.86	OS,PFS	8
Wan et al.(2)	2022	China	Cohort(retrospective)	65	2018-2021	55	15.3	Anti-PD-1+ chemo	combined therapy	1st-line	Baseline	Univariate	2.86	OS,PFS	8
Yuan et al.	2022	China	Cohort(retrospective)	59	2016-2021	357	NA	Anti-PD-(L)1	Monotherapy, combined therapy	1st, or 2nd, or 3rd line immunotherapy	Baseline	Univariate(OS)/Multivariate(PFS)	NA	OS,PFS	7

GC, gastric cancer;NOS, Newcastle–Ottawa scale; OS, overall survival; PFS, progression-free survival; LMR, lymphocyte/monocyte ratio; ICIs, immune checkpoint inhibitors; PD-(L)1, programmed death- (ligands) 1; CTLA-4, cytotoxic T lymphocyte antigen 4; chemo, chemotherapy. NA, not available.

### 3.2 Study quality

All eight studies scored between 7 and 8 on the NOS scale, indicating high quality (**Supplementary Table S2**).

### 3.3 Meta-analysis results

#### 3.3.1 LMR and OS

**Figure 2:**
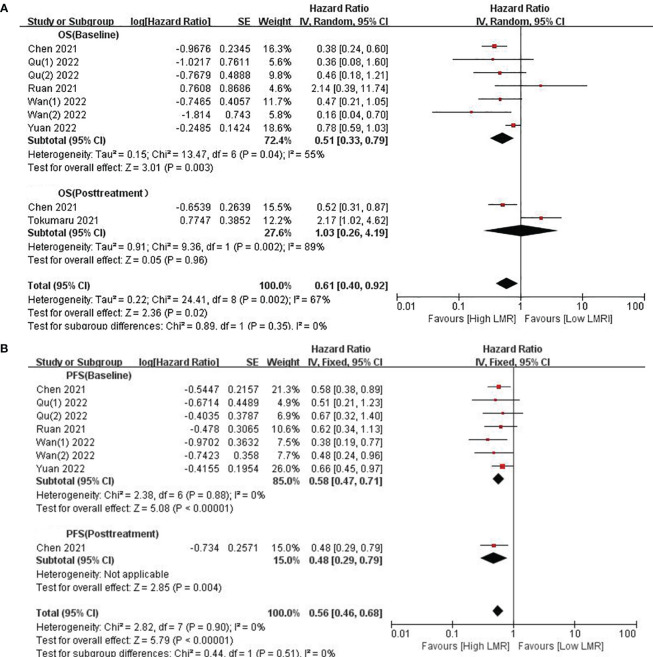
**(A)** Forest plots for the association between LMR and OS; **(B)** Forest plots for the association between LMR and PFS.

We investigated the relationship between LMR and OS, involving eight cohort studies comprising 815 participants. Among these studies, six provided baseline LMR values exclusively, one offered post-treatment LMR data only, and one provided both baseline and post-treatment LMR values. Given the substantial heterogeneity amongst related studies (*I* ^2^ = 67%, p=0.002), a random-effects model was adopted (**Figure 2A**). The results revealed a significant and favorable correlation of high LMR values with prolonged OS in GC patients receiving ICI therapy (HR=0.61, 95% CI: 0.40–0.92; p=0.02, **Figure 2A**). Subgroup analysis was conducted based on the timing of LMR measurement. The analysis results demonstrated an association between high baseline LMR values and improved OS (HR=0.51, 95% CI: 0.33–0.79; p=0.003, **Figure 2A**), and there was significant heterogeneity (*I* ^2 = ^55%, p=0.04). No significant association was found between LMR and OS after treatment (HR=1.03, 95% CI: 0.26–4.19; p=0.96, **Figure 2A**).

Given that only two studies provided post-treatment LMR data, limiting the feasibility of subgroup analysis, we proceeded with stratified analysis based on baseline LMR. To detect potential heterogeneity, we conducted subgroup analyses by sample size, follow-up time, drug combination, line of therapy, and cut-off values. **Table 2** showcases the outcomes of these analyses. Firstly, in studies with a sample size < 100 (HR: 0.46; 95% CI: 0.25–0.84; p=0.01), the high-LMR group exhibited a significantly improved OS. Conversely, in studies with a sample size ≥100, no significant prognostic effect of LMR was found (HR=0.56, 95% CI: 0.28–1.13; p=0.1). Secondly, subgroup analysis based on follow-up time revealed a significantly better OS in the high-LMR group among patients followed for over 16 months compared to those receiving shorter duration of follow-up (HR=0.40, 95% CI: 0.27–0.59; p<0.00001). Thirdly, the subgroup analysis based on types of immunotherapy unveiled no significant prognostic effect of either monotherapy (P=0.38) or the combination of different ICIs (P=0.1). However, significantly improved OS was observed in participants with high LMR and who received combination therapy (HR: 0.40; 95% CI: 0.23–0.67; p=0.0006). In addition, a high LMR in GC patients receiving first-line treatment (HR=0.37; 95% CI: 0.20–0.69; p=0.002) was an important prognostic factor for favorable OS compared to GC patients receiving second-line treatment (P=0.07). Furthermore, a high LMR cut-off (≥3.0) was predictive of increased OS in GC patients (HR=0.39, 95% CI: 0.26–0.58; p<0.00001).

**Table 2:** Pooled HRs for OS and PFS in subgroup analyses.

Subgroup	OS (Baseline)			PFS (Baseline)				
	Study	HR [95%CI]	P value	I2	Study	HR [95%CI]	P value	I2
***Total***	7	0.51 [0.33-0.79]	0.003	55%	7	0.58 [0.47-0.71]	<0.00001	0%
Sample size								
≥100	2	0.56 [0.28-1.13]	0.1	85%	2	0.62 [0.47-0.83]	0.0001	0%
<100	5	0.46 [0.25-0.84]	0.01	23%	5	0.53 [0.38-0.72]	0.001	0%
Follow-up								
≥16months	3	0.40[0.27-0.59]	<0.00001	0%	3	0.52 [0.37-0.72]	0.0001	0%
<16months	3	0.51 [0.15-1.75]	0.28	61%	3	0.58 [0.40-0.86]	0.007	0%
Combined medication								
Monotherapy	1	2.14 [0.39-11.74]	0.38	NA	1	0.62 [0.34-1.13]	0.12	NA
Combined therapy	4	0.40 [0.23-0.67]	0.0006	0%	4	0.49 [0.34-0.72]	0.0002	0%
Monotherapy+combined therapy	2	0.56 [0.28-1.13]	0.1	85%	2	0.62 [0.47-0.83]	0.001	0%
Line of therapy								
1st-line	3	0.37 [0.20-0.69]	0.002	0%	3	0.44 [0.29-0.69]	0.0003	0%
other	4	0.61 [0.35-1.05]	0.07	68%	4	0.63 [0.49-0.80]	0.0002	0%
LMR cut-off								
LMR≥3	3	0.39 [0.26-0.58]	<0.00001	61%	3	0.59 [0.42-0.82]	0.002	0%
LMR<3	3	0.51 [0.16-1.66]	0.26	0%	3	0.50 [0.34-0.73]	0.0004	0%

OS, overall survival; PFS, progression-free survival; HR, hazard ratio; CI, confidence interval. NA, not available.

#### 3.3.2 LMR and PFS

Eight studies contributed data on LMR and PFS, encompassing six articles providing baseline LMR values and one study providing baseline and post-treatment LMR. Echoing the findings from our analysis of OS, elevated LMR was found to be correlated with prolonged PFS in GC patients on ICIs (HR=0.56, 95% Cl: 0.46–0.68;p<0.00001, **Figure 2B**), with no substantial heterogeneity observed (*I* ^2^ = 0%, p=0.90). Similarly, a higher baseline LMR was linked to enhanced PFS (HR=0.58; 95% CI: 0.47–0.71; p<0.00001, **Figure 2B**), with no significant heterogeneity observed (*I* ^2^ = 0%, p=0.88). Only one study reported on patients with higher LMR after treatment (HR=0.48; 95% CI: 0.29–0.79; p=0.004, **Figure 2B**), and the results indicated that higher LMR after treatment was correlated with prolonged PFS.

Therefore, our subgroup analyses concentrated solely on baseline LMR. These analyses revealed the association between high levels of baseline LMR and improved PFS, but no significant prognostic effect was observed with monotherapy (P=0.12). Across various parameters, including sample size, follow-up time, combination of drugs, line of treatment, and cut-off value, elevated LMR was consistently correlated with improved PFS (p<0.05; **Table 2**), and there was no significant heterogeneity (I^2^ = 0%).

### 3.4 Sensitivity analysis

**Figure 3:**
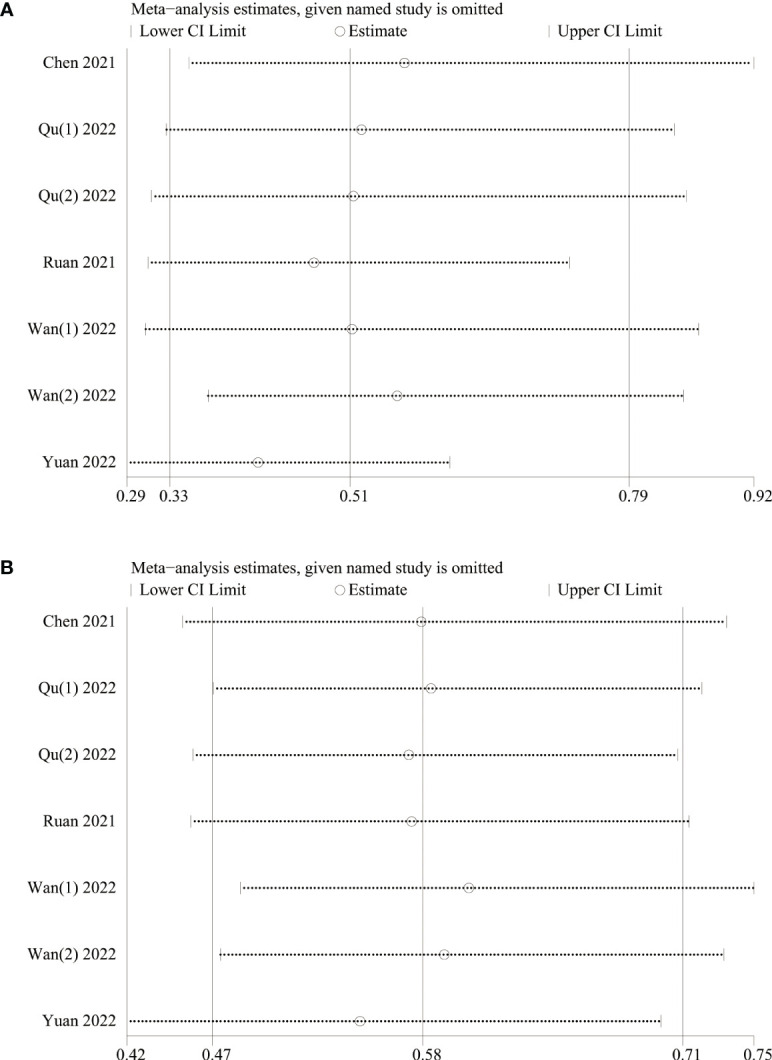
Sensitivity analysis of **(A)** OS and **(B)** PFS.

A sensitivity analysis was conducted to examine the robustness of our analysis results associated with the clinical significance of baseline LMR, revealing that the effect size remained consistent within the original range after each study was sequentially removed. This indicated that no single study disproportionately influenced the outcomes for both OS (**Figure 3A**) and PFS (**Figure 3B**), confirming the reliability of analysis results.

### 3.5 Publication bias

**Figure 4:**
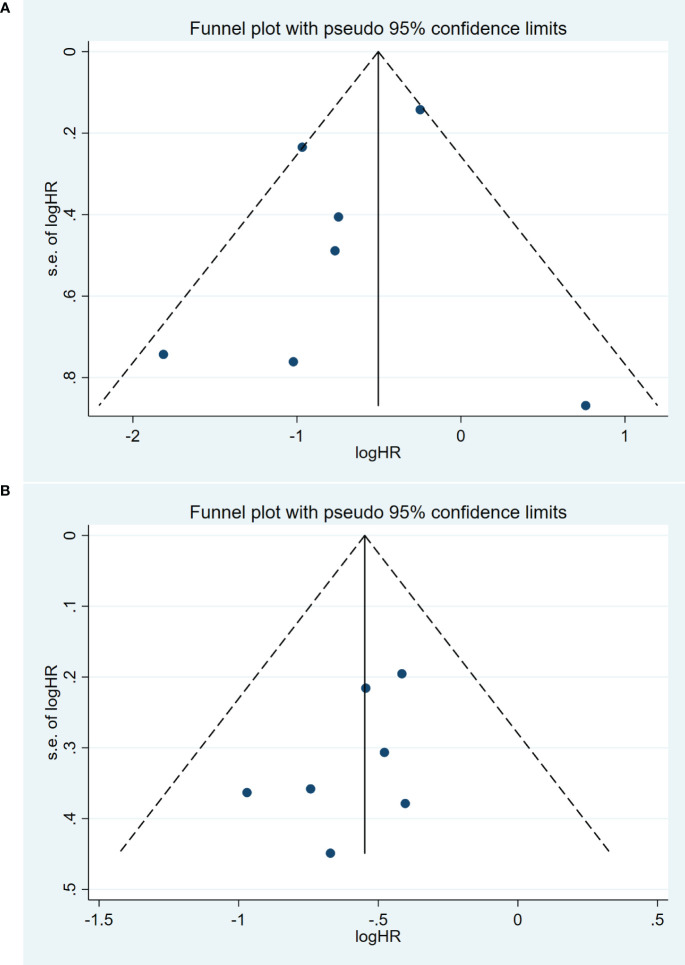
Funnel plot for the evaluation of publication bias for **(A)** OS and **(B)** PFS.

Publication bias was assessed using a funnel plot and Egger’s test. The symmetrical funnel plot suggested the absence of substantial publication bias in the meta-analysis concerning OS (Egger: p=0.36) (**Figure 4A**). The Egger’s test results also indicated no significant publication bias in the meta-analysis for PFS (Egger: p=0.19) (**Figure 4B**). However, we were unable to conduct a publication bias analysis for the remaining studies due to the limited number of studies (<3 studies).

## 4 Discussion

Throughout the various stages of tumor development, systemic inflammation emerges as a central player, influencing genetic mutations, genomic instability, epigenetic alterations, tumor metastasis, and the proliferation of cancer cells (28, 29). Blood-derived parameters offer a readily accessible and reproducible means of assessing systemic inflammation, serving as objective biomarkers to predict patient prognosis (30, 31). However, the evidence linking a high LMR with improved survival outcomes in these cancers has been somewhat limited (32). Initially conceived as a prognostic indicator for hematologic malignancies, the pre-treatment LMR has been demonstrated in numerous studies to reflect systemic inflammation and positively correlate with the prognoses of various solid neoplasms, such as melanoma, breast cancer, and GC (19, 20, 24–27, 33–35). Nevertheless, the precise mechanism underlying the prognostic impact of LMR on GC remains elusive. LMR hinges on the levels of lymphocytes and monocytes, which serve as indicators of anti-tumor immunity and tumor burden (36). Low lymphocyte counts can result in an impaired immune response against cancer cells, particularly tumor-infiltrating lymphocytes (TILs), which are pivotal for the cell-mediated anti-tumor immune response (37). Furthermore, increased TILs are associated with improved outcomes in cancer patients (38). The infiltration of CD4 T cells triggers the activation of CD8 T cells, instigating apoptosis and cytotoxic activity against cancer cells (39, 40). Consequently, diminished lymphocyte counts may contribute to reduced survival rates across various cancers (41, 42). In addition to lymphocytes, blood monocytes hold the capacity to differentiate into tumor-associated macrophages (TAMs) that play a crucial role in tumorigenesis. TAMs directly affect regulatory T-cells, impeding tumor immunity while accelerating angiogenesis and the degeneration of the extracellular matrix. These actions promote the development and progression of tumors (43, 44). As a result, TAMs can be considered an indicator of a high cancer burden. Thus, LMR is regarded as a reflection of the immune state and holds the potential to function as a prognostic indicator for the effectiveness of ICIs.

This meta-analysis involved 815 patients and aimed to evaluate the prognostic significance of LMR in GC patients on ICIs. Significant positive associations were observed between baseline LMR and OS, as well as between OS and PFS. It’s worth noting that only one study in our analysis reported an improvement in PFS for patients with a high LMR after treatment. However, the correlation between OS and post-treatment LMR was not as evident. This discrepancy could be attributed to the scarcity of studies providing data on post-treatment LMR and the variability in the timing of LMR assessments, spanning from 2 to 6 weeks after the initial dosage. Evidence has shown the “true” mobilization time of activated white blood cells into the bloodstream typically requires at least 4 weeks (45). This observation may help clarify the inconsistent conclusions observed in articles reporting on post-treatment LMR. Therefore, future research could examine whether variations in the timing of post-treatment LMR assessment indeed impact clinical outcomes and whether changes in LMR before and after ICI administration correlate with patient prognosis. In our efforts to provide a more nuanced analysis, we conducted subgroup assessments of various treatments to explore the relationship between a high LMR and both OS and PFS. Our analysis results were deemed reliable following the publication bias test. To the best of our knowledge, this meta-analysis is the initial endeavor to examine the predictive significance of LMR in GC patients who received ICI treatment. Our findings bear significant implications for the clinical management of GC patients undergoing PD-1/PD-L1 antibody treatment. Specifically, our analysis results suggest that GC patients with a low LMR before treatment may face an elevated risk of cancer progression or relapse following initial chemotherapy. This underscores the importance of close monitoring and follow-up for these patients. Notably, in the study by Ruan et al. (25), the univariate analysis revealed that similar predictive performance of LMR for OS compared to other studies (HR=0.27, 95% CI: 0.12-0.61), whereas the multivariate analysis unraveled opposite results on OS(HR=2.14, 95%CI: 0.39-11.69).

Meta-analyses of various studies have consistently highlighted the promising prognostic potential of LMR in specific cancer patient populations undergoing ICI treatment. For instance, a recent meta-analysis that encompassed 21 studies illuminated the potential of a high pre-treatment LMR as a robust prognostic biomarker for advanced cancer patients receiving immunotherapy (46). Our meta-analysis, wherein we amalgamated data from 815 patients, affirmed the significant prognostic significance of LMR in GC patients on ICIs. This finding aligns with previous prior research on the prognostic significance of LMR in diverse tumor types.

Our meta-analysis, albeit informative, does come with some limitations that warrant consideration. Firstly, all eligible studies in our analysis were conducted in Asia, specifically China and Japan. Consequently, our conclusions should be interpreted within this geographical context, and prudence is warranted when extrapolating our findings to patients residing in Europe, Africa, the Americas, and other regions. Indeed, additional research is necessary to validate the prognostic relevance of LMR in non-Asian GC patients undergoing ICI treatment. Secondly, the majority of the studies included in our analysis adopted a retrospective design rather than a prospective one. This retrospective nature introduces the potential for confounding factors that may influence the reliability of our results. Furthermore, the variability in LMR cut-off values employed across the included studies is another limitation. These cut-off values ranged from 2.8 to 5, potentially introducing inherent heterogeneity into our meta-analysis due to data inconsistencies. To foster greater reliability and comparability in future studies, it is essential that researchers establish a standardized cut-off value for LMR.

In summary, our meta-analysis reveals that high pre-treatment LMR is significantly correlated with favorable outcomes, such as prolonged OS and PFS, in GC patients treated with ICIs. This indicates that LMR could serve as an independent and informative prognostic biomarker for GC, thereby aiding in making informed treatment decisions concerning immunotherapy for GC. However, given the limitations inherent in the studies incorporated into our analysis, additional prospective trials are required to substantiate our findings across diverse ethnicities and geographical regions.

## Data availability statement

The original contributions presented in the study are included in the article/**Supplementary Material**. Further inquiries can be directed to the corresponding authors.

## Author contributions

PM: Conceptualization, Data curation, Formal analysis, Methodology, Software, Writing – original draft. WF: Conceptualization, Investigation, Supervision, Writing – review & editing. YZ: Data curation, Methodology, Supervision, Writing – review & editing. XG: Funding acquisition, Project administration, Resources, Supervision, Writing – review & editing.
