# Supplementary material for: Prognostic value of lymphocyte-to-monocyte ratio in gastric cancer patients treated with immune checkpoint inhibitors: a systematic review and meta-analysis
Source: Front Immunol. 2023 Nov 27;14:1321584. doi: 10.3389/fimmu.2023.1321584 (PMC10711042; doi:10.3389/fimmu.2023.1321584)
Supplement: Supplementary file 1 [file Table_1.docx]

| Supplementary Table S1. Detailed search strategy in four databases. | |
| --- | --- |
| Database | Search strategy |
| Pubmed | (((Lymphocytes) AND (Monocytes)) AND (ratio))) AND(Immune Checkpoint Inhibitors) AND(Stomach Neoplasms) |
| Embase | 1 stomach tumor  2 immune checkpoint inhibitor  3 monocyte lymphocyte ratio  4 lymphocyte monocyte ratio  5 3 or 4  6 1 and 2 and 5 |
| Web of Science | #1 Cancer of Stomach (Topic) or Stomach Neoplasms (Topic) or tumour of the stomach (Topic)  #2 Checkpoint Inhibitors, Immune (Topic) or immune checkpoint inhibitor (Topic) or Nivolumab (Topic)  #3 monocyte to lymphocyte ratio (Topic) or lymphocyte to monocyte ratio (Topic)  #4 #1 AND #2 AND #3 |
| Chochrane | #1 Stomach Neoplasms  #2 Immune Checkpoint Inhibitors  #3 (Lymphoid Cells) OR (Cell, Lymphoid) OR (Cells, Lymphoid)  #4(monocytes) OR (monocytes, activated killer)  #5 ratio  #6 #3 and #4 and #5  #7 #1 and #2 and #6 |
