# Supplementary material for: Prognostic value of lymphocyte-to-monocyte ratio in gastric cancer patients treated with immune checkpoint inhibitors: a systematic review and meta-analysis
Source: Front Immunol. 2023 Nov 27;14:1321584. doi: 10.3389/fimmu.2023.1321584 (PMC10711042; doi:10.3389/fimmu.2023.1321584)
Supplement: Supplementary file 2 [file Table_2.docx]

| Supplementary Table S2. Quality evaluation of the eligible studies with Newcastle–Ottawa scale. | | | | | | | | | |
| --- | --- | --- | --- | --- | --- | --- | --- | --- | --- |
| Study | Selection | | | | Comparability | | Outcome | | |
|  | Representative-ness | Selection of  non-exposed | Ascertainment  of exposure | Outcome not present at start | Comparability on most important factors | Comparability on other risk factors | Assessment of outcome | Long enough follow-up (median≥1 year) | Adequacy  (completeness) of follow-up |
| Chen et al. | * | * | * | * | - | - | * | * | * |
| Qu et al.(1) | * | * | * | * | * | - | * | * | * |
| Qu et al.(2) | * | * | * | * | * | - | * | * | * |
| Ruan et al. | * | * | * | * | * | - | * | - | * |
| Tokumaru et al. | * | * | * | * | * | - | * | * | * |
| Wan et al.(1) | * | * | * | * | * | - | * | * | * |
| Wan et al.(2) | * | * | * | * | * | - | * | * | * |
| Yuan et al. | * | * | * | * | - | - | * | * | * |
| *indicates criterion met; - indicates significant of criterion not met. | | | | | | | | | |
|  | | | | | | | | | |
